# Improved Mental Acuity Forecasting with an Individualized Quantitative Sleep Model

**DOI:** 10.3389/fneur.2017.00160

**Published:** 2017-04-25

**Authors:** Brent D. Winslow, Nam Nguyen, Kimberly E. Venta

**Affiliations:** ^1^Design Interactive Inc., Orlando, FL, USA

**Keywords:** sleep, machine learning, actigraphy, cognition, mobile applications, executive function

## Abstract

Sleep impairment significantly alters human brain structure and cognitive function, but available evidence suggests that adults in developed nations are sleeping less. A growing body of research has sought to use sleep to forecast cognitive performance by modeling the relationship between the two, but has generally focused on vigilance rather than other cognitive constructs affected by sleep, such as reaction time, executive function, and working memory. Previous modeling efforts have also utilized subjective, self-reported sleep durations and were restricted to laboratory environments. In the current effort, we addressed these limitations by employing wearable systems and mobile applications to gather objective sleep information, assess multi-construct cognitive performance, and model/predict changes to mental acuity. Thirty participants were recruited for participation in the study, which lasted 1 week. Using the Fitbit Charge HR and a mobile version of the automated neuropsychological assessment metric called CogGauge, we gathered a series of features and utilized the unified model of performance to predict mental acuity based on sleep records. Our results suggest that individuals poorly rate their sleep duration, supporting the need for objective sleep metrics to model circadian changes to mental acuity. Participant compliance in using the wearable throughout the week and responding to the CogGauge assessments was 80%. Specific biases were identified in temporal metrics across mobile devices and operating systems and were excluded from the mental acuity metric development. Individualized prediction of mental acuity consistently outperformed group modeling. This effort indicates the feasibility of creating an individualized, mobile assessment and prediction of mental acuity, compatible with the majority of current mobile devices.

## Introduction

Sleep is a well-conserved physiological state behaviorally characterized by reduced motor activity and response to stimulation, easy reversibility, stereotypic postures, and characteristic patterns of brain activity. Sleep duration and quality significantly alters brain structure and function. For instance, brain-related changes associated with sleep include alterations to hippocampal function (1), metabolic clearance (2), neuroendocrine function (3), and formation of dendritic spines (4). Sleep impairment is known to cause learning dysfunction (5), performance degradations (6), and is associated with depression (7), and impaired physical health (8). Sleep disturbances are common across multiple professions, and the proportion of individuals reporting short sleep duration (<6 h per night) is increasing (9), along with accompanying medical problems (10). For example, during military deployment, poor sleep health is common due to hazardous work conditions, inconsistent hours, crowded sleep spaces, harsh environments, travel across time zones, and exposure to noise (11). Following deployment, sleep disturbance affects a high number of military veterans and is associated with mental illness (12).

A growing body of research has sought to model the relationship between sleep and cognitive performance (13). Early efforts included the three process model of alertness/performance (TPM), developed to predict group performance and alertness throughout a day (14). The more recent unified model of performance expands on the TPM and more closely models individual psychomotor performance variance (15–18). In order to use the previous models to predict individual cognitive performance using supervised learning techniques, actual cognitive performance data, usually provided by the psychomotor vigilance test (PVT), is required to update the model–parameter estimates (19). The PVT is a simple reaction time exam sensitive to the effects of fatigue and sleepiness (20). It requires subjects to respond to a rare, random stimulus with an interstimulus interval typically between 2 and 12 s over a 5–10 min session. Model inputs from the PVT include the number of lapses, defined as misses or responses exceeding a pre-defined temporal threshold (19), and average response times (RTs) over a PVT session (21). However, psychomotor vigilance is only one aspect of changes to cognitive function associated with sleep. There is a need for models that implement and predict expanded aspects of cognitive function.

Previous modeling efforts have relied on self-reported sleep metrics such as sleep duration, bedtime, and sleep quality as model inputs. Available evidence suggests that many individuals systematically under- or overestimate their sleep habits (22). Given that accurate sleep information is critical to modeling sleep-related circadian changes to mental acuity, there is also a need for models that implement objective sleep features. Wearable systems are rapidly expanding (23) and many include algorithms designed to estimate an individual’s wakefulness, restlessness, and sleep *via* multi-axis accelerometry. Such algorithms identify the shift from high activity during wake to relativity low activity during sleep. Wearable systems continue to evolve by including additional sensors such as pulse photoplethysmography (PPG) to assess heart rate, which may have added utility in sleep monitoring (24).

The objective of the current study was to develop algorithms and a mobile application to quantify and predict sleep-related changes to cognitive functions including working memory, reaction time, executive function, and psychomotor vigilance using wearable technology. We hypothesize that individualized mental acuity modeling will outperform group modeling and that an expanded mental acuity metric, utilizing vigilance, working memory, and linguistic capability will be subject to circadian alteration and reduction following sleep loss.

## Materials and Methods

### Participants

Thirty adult participants were recruited from the Orlando, FL, USA area for participation in the study, which lasted 1 week. Participants were recruited using recruitment flyers posted online and through recruitment fairs located at local universities. Inclusion criteria included age (18–30 years), good general health, no self-reported sleep disorders, no medications that may affect sleepiness, and normal or corrected-to-normal vision. In order to maintain participant confidentiality, all participants were assigned a unique three digit code, from A01 to A30, based on order of inclusion.

### Materials

The Charge HR (Fitbit, San Francisco, CA, USA) was used to quantify metrics associated with sleep and activity. Fitbit data, including sleep, activity, and heart rate, was sent to mobile phones *via* Bluetooth. Assessment of mental acuity was accomplished using CogGauge (25)—a mobile suite of assessments based on the automated neuropsychological assessment metrics [ANAM^®^ (26)]. Specific assessments implemented included: mathematical processing, which assesses working memory by requiring the subject to perform basic arithmetic operations (Pearson correlation coefficient = 0.87); PVT, which assesses reaction time and vigilance by requiring the subject to respond to a stimulus that appears between 2 and 12 s random interval (PCC = 0.81); running memory continuous performance test, a 1-Back test, which assesses working memory (PCC = 0.80); and logical reasoning, a linguistic task requiring the comprehension of simple statements and grammatical transformations (PCC = 0.69) (27). The PVT lasted 5 min per session; the other assessments lasted 1–2 min each per session (Table 1).

**Table 1:** **Mobile assessment suite based on the automated neuropsychological assessment metric**.

Assessment	Description	Interface
Psychomotor vigilance	Reaction time and vigilance assessed over 5 min. Subject responds as quickly as possible when a stimulus appears	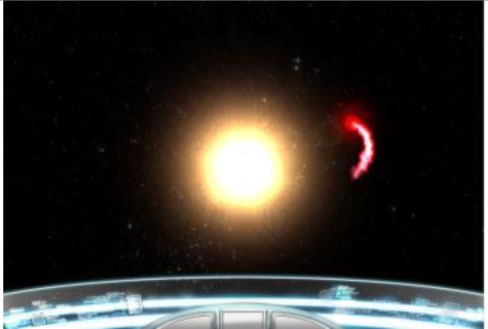
Logical reasoning	Linguistic task requiring the subject to determine whether various grammatical relationships describe the order of 2+ objects	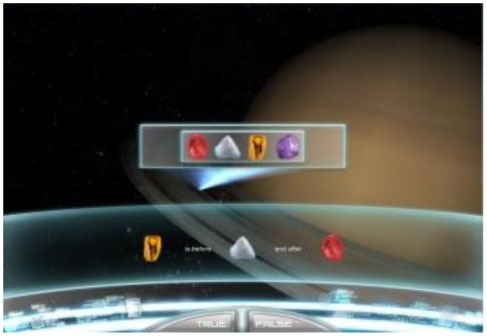
Math processing	Working memory assessment requiring the subject to quickly perform basic arithmetic operations	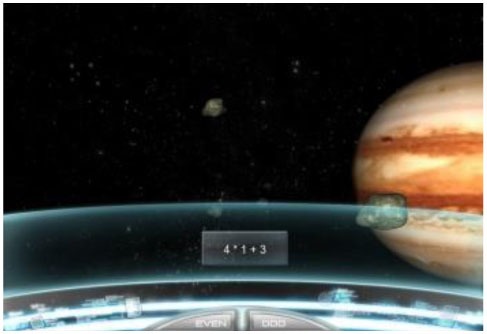
1-Back	Requires subjects to recall the last digits that appeared on the screen and decide if the current digits displayed are different	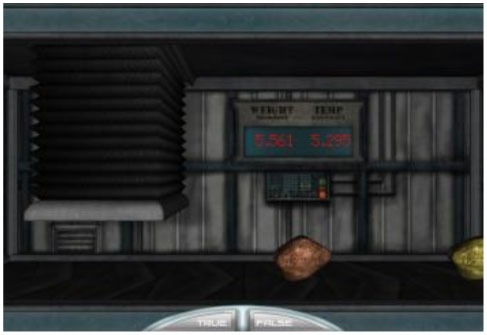

A custom application was also created to allow participants to input caffeine consumption. Actigraphy data, caffeine consumption, and CogGauge data were implemented on mobile phones, including the Motorola Moto-X second generation running Android 6.0, the Samsung Galaxy S5 (SGS5) running Android 5.0, the iPhone 5 and the iPhone 6+ running iOS 9.3.2.

### Experimental Procedure

Upon arrival, participants provided written informed consent, responded to a demographics questionnaire and sleep scales. Participants were issued an actigraphy system and a mobile phone for data collection. Participants were instructed to wear the actigraphy device throughout the week with the exception of swimming or bathing, and to respond to the CogGauge assessments during convenient awake hours, three times daily. Notification reminders were included in the mobile application to request CogGauge assessments be taken at a random time within three intervals: morning 9:00 a.m.–12:00 p.m., afternoon 1:00–5:00 p.m., and evening 5:00–9:00 p.m. Verbal instructions were also given to vary the times of testing in order to sample from their entire circadian rhythm. Participants also tracked their caffeine consumption in the custom application. After the 7-day data collection, participants returned the actigraphy system and phone, and were debriefed and paid $160 for their participation.

### Subjective Measures

During the initial session with the participant, a number of subjective measures were provided electronically. The Stanford Sleepiness Scale was used to assess current alertness (28). The Epworth Sleepiness Scale was also used to assess subject’s general level of daytime sleepiness (29). Self-reported sleep habits (average number of hours of sleep per night) were provided by participants *via* self-report.

### Objective Measures

The Fitbit API (http://dev.fitbit.com) provided access to features used for modeling in 1-min increments including heart rate and activity including number of steps, flights of stairs, and types of physical activity, as well as aggregated measures of sleep events such as minutes asleep, restless, and awake, and sleep start times and durations.

Several features were extracted from the CogGauge battery and are listed in Table 2. Feature values were only calculated for assessments with responses to over five questions. RT was defined as the time a participant took to respond after presentation of a question. Correct response time (CRT) represents the RT of questions answered accurately. Questions that were not answered in the allotted time were considered timeouts, and their RT values were not included in the calculation of statistical values for RT.

**Table 2:** **Mental acuity metrics included in feature analysis for each assessment**.

Measurement	Value	Psychomotor vigilance	Logical reasoning	Math processing	1-Back
Response times	Mean	X	X	X	X
	Median	X	X	X	X
	SD	X	X	X	X

Correct response time (CRT)	Mean	X	X	X	X
	Median	X	X	X	X
	SD	X	X	X	X

Correct responses	Count	X	X	X	X
	Rate	X	X	X	X
	Percentage	X	X	X	X

Incorrect responses	Count	X	X	X	X

Timeout	Count	X	X	X	X

Lapse	Count	X			

Distracted answer	Count	X			

Misfire	Count	X			

*Included metrics are marked with X*.

Psychomotor vigilance test feature definitions differed from the other three assessments. While the logical reasoning, math processing, and 1-Back assessments presented participants with questions that had correct and incorrect answers, PVT questions only required a response to a stimulus. If a participant responded before the stimulus, the answer was considered a misfire. For PVT, incorrect responses were defined as any questions whose response was either a timeout or a misfire, defined as a response prior to stimulus. Lapses were defined as PVT questions with RTs greater than 500 ms. Distracted answers were defined as responses over 1,250 ms.

### Participant Compliance

Participants were asked to respond to a complete session of CogGauge, consisting of four assessments, three times daily. A complete session was defined as 20 questions each of the 1-Back, logical reasoning, and math processing tests, as well as 5 min of PVT. Compliance was measured as each participant’s percent deviation from 100% use. Participants were not expected to wear their Fitbit continuously for the entire week due to removal recommendations for bathing and charging requirements. Ideal compliance for the time the Fitbit was worn was measured as continuous wear with time off wrist of no more than 3.5 h (a single 1.75 h charging session plus 15 min daily for bathing). Non-zero Fitbit heart rate data were used to define the periods of time the Fitbit was worn. The number of main sleep events the Fitbit logged was also measured for each user, with ideal compliance represented by seven main sleep events during the week, to ensure sleep events were recording properly.

### Data Modeling

The feature list in Table 2 was reduced to create an uncorrelated feature space of the most descriptive features that would operate consistently across phone models where differences in RTs are expected due to differences in hardware capabilities. To accomplish this, the variability in RT measurements between phone models was addressed by disregarding any features that rely on the absolute value of the RT. Pearson’s correlation coefficient was calculated for each pair of features. Features with known correlations were eliminated. Finally, an exhaustive wrapper feature importance measurement was performed to determine the most influential features for predicting time asleep. For this process, a cross-validated linear regression model was trained for all possible subsets of the features, and the Bayes Information Criterion (BIC) and root mean squared error (RMSE) was measured. While the RMSE measures how well the model fits the data, the BIC additionally incorporates a penalty term for the inclusion of extra features to avoid unnecessary features and overfitting (30). The final features selected for input into the model were chosen as the feature set with the lowest BIC that still represented at least three games, to ensure a broad definition of mental acuity, and at least one measure from PVT, to maintain consistency with previous models.

An overall mental acuity metric was designed as an equally weighted linear combination of all selected features set on an approximate 0–100 scale, where 0 represents no responses to any CogGauge assessments and 100 represents exceptional performance across all features. Unbounded variables, such as SD in RT, were scaled with a value double the λ value from a Poisson fit to the distribution of values.

### Data Analysis and Statistics

A Kruskal–Wallis test was performed between phone models on all RTs, as defined above, to determine if RT varied between phone models.

The predictive model was designed to fit the unified model of performance to the mental acuity metric (dependent variable) and the sleep records (independent variable) using the method of least squares. Vertical translation and scaling terms were added to convert the unified model of performance output to the mental acuity metric scale. The final form of the equation used in the model fit is given in Eq. 1:

(1) $$\begin{array}{l} \text{Mental Acuity Metric}=v+\beta \left[0.97\;\sin\left(\frac{2\pi }{\tau }\left(t-t_{w}+\varphi \right)\right)\right]+\\ \;\alpha \left\{ \begin{array}{l} \;S_{0}e^{-\left(t-t_{0}\right)/\tau_{w}}+U\left(1-e^{-\left(t-t_{0}\right)/\tau_{w}}\right),\text{while awake}\\ \;S_{0}e^{-\left(t-t_{0}\right)/\tau_{s}}-2U\left(1-e^{-\left(t-t_{0}\right)/\tau_{s}}\right)\\ \;+\frac{\left(L_{0}-2U\right)\tau_{\text{LA}}}{\tau_{\text{LA}}-\tau_{s}}\left(e^{-\left(t-t_{0}\right)/\tau_{\text{LA}}}-e^{-\left(t-t_{0}\right)/\tau_{s}}\right),\text{while asleep} \end{array}\right. \end{array}$$

where *v* is the vertical shift term, β is the circadian rhythm scaling term, τ is the period of the circadian rhythm, *t* is the current time, *t_w_* is the most recent waking time, φ is the offset of the circadian rhythm relative to waking time, α is the homeostatic process scaling term, *t*_0_ is the time of the most recent transition between sleeping and waking, *S*_0_ is the homeostatic state at *t*_0_, *U* is the upper limit of a sleep reservoir, τ*_w_* is the time constant of the homeostatic process during waking, τ*_s_* is the time constant of the homeostatic process during sleeping, *L*_0_ is the lower limit of a sleep reservoir at *t*_0_, and τ_LA_ is an additional time constant of the unified model of performance. *L*_0_ was calculated from the *L*_0_ of the previous period of sleeping or waking, *L*_0,previous_, and previous *t*_0_, *t*_0,previous_, *via* Eq. 2:

(2) $$L_{0}=\left\{ \begin{array}{l} \;U+\left(L_{0,\;\text{previous}}-U\right)e^{-\left(t_{0}-t_{0,\;\text{previous}}\right)/\tau_{\text{LA}}}\\ \;L_{0,\text{previous}}e^{-\left(t_{0}-t_{0,\;\text{previous}}\right)/\tau_{\text{LA}}}\\ \;-2U\left(1-e^{-\left(t_{0}-t_{0,\;\text{previous}}\right)/\tau_{\text{LA}}}\right),\;\text{while asleep} \end{array}\right.$$

*t, t_w_*, and *t*_0_ were determined from Fitbit data. The time constants were fixed at τ*_w_* = 10 h, τ*_s_* = 2 h, and τ_LA_ = 166 h, as modification of their values had minimal effect on the fit quality of the model (16). All remaining parameters (*y*, β, τ, φ, α, *S*_0_, *U*, and *L*_0_) were optimized *via* the least squares fit to the model.

Two models were trained on the mental acuity and sleep data. The first was a group model trained across all participants’ data to predict the mental acuity of a generalized user. The second was a set of individual models trained on a specific participant’s data to predict the participant’s unique response to sleep. The group model was cross validated with three iterations by holding out a random participant’s data for testing during each iteration. The individual models were cross validated with three iterations each by holding out a random day’s data for testing during each iteration.

The ultimate goal of this effort was to create an individualized model of the effects of sleep on mental acuity. In order to measure model performance, the following equation was used:

$$\text{fit quality}={\text{SD}}_{\text{mental acuity}}{\text{/RMSE}}_{\text{UMP}}$$

where SD_mental acuity_ is the standard deviation over all mental acuity values used in the model and RMSE_UMP_ is the RMSE of the unified model of performance fit to the data. This measure represents a model that is no different than a measure of mental acuity independent of sleep effects, and the larger the value greater than one, the better the improvement of the UMP over such a model. This fit quality was calculated for both group and individual models.

## Results

The sociodemographic factors in the study sample are listed in Table 3. Most participants were male, of an average age 23.2 ± 3.88 years, and reported sleeping 7.3 ± 0.64 h per night. The mean Epworth Sleepiness Scale score of 6.23 ± 2.47 indicated the study sample to be average compared to population norms (31).

**Table 3:** **List of sociodemographic factors in the study sample**.

	Study sample% (*n*)
**Gender**	
Male	63.3 (19)
Female	36.7 (11)
**Age group**	
18–21	43.3 (13)
22–25	30.0 (9)
26–30	26.7 (8)
**Education**	
High school diploma/GED	46.7 (14)
Some college/university	30.0 (9)
University degree	23.7 (7)
**Average self-reported sleep duration**	
4–6 h	3.3 (1)
6–8 h	63.3 (19)
>8 h	33.3 (10)
**Epworth Sleepiness Scale**	6.23 ± 2.47 (SD)

Four users had sleep events manually defined based on heart rate and actigraphy data. Two users had continuous heart rate data for the duration of the study, indicating they were wearing the device, but no records of sleep events for periods of 3 days or more. Two additional users had intermittent heart rate data.

Less severe heart rate tracking issues caused some sleep events to become fragmented, where Fitbit reported a new sleep event beginning as little as 1 min after the previous event ended. These events were combined if the time awake between events was less than 1.5 h, or if the time awake between events was less than 3 h and fewer than 85% of those minutes included heart rate data. Minutes awake for the intervening time were calculated as the number of minutes where the device recorded steps, indicative of participant ambulatory movement, and all other intervening minutes were considered minutes asleep.

**Figure 1:**
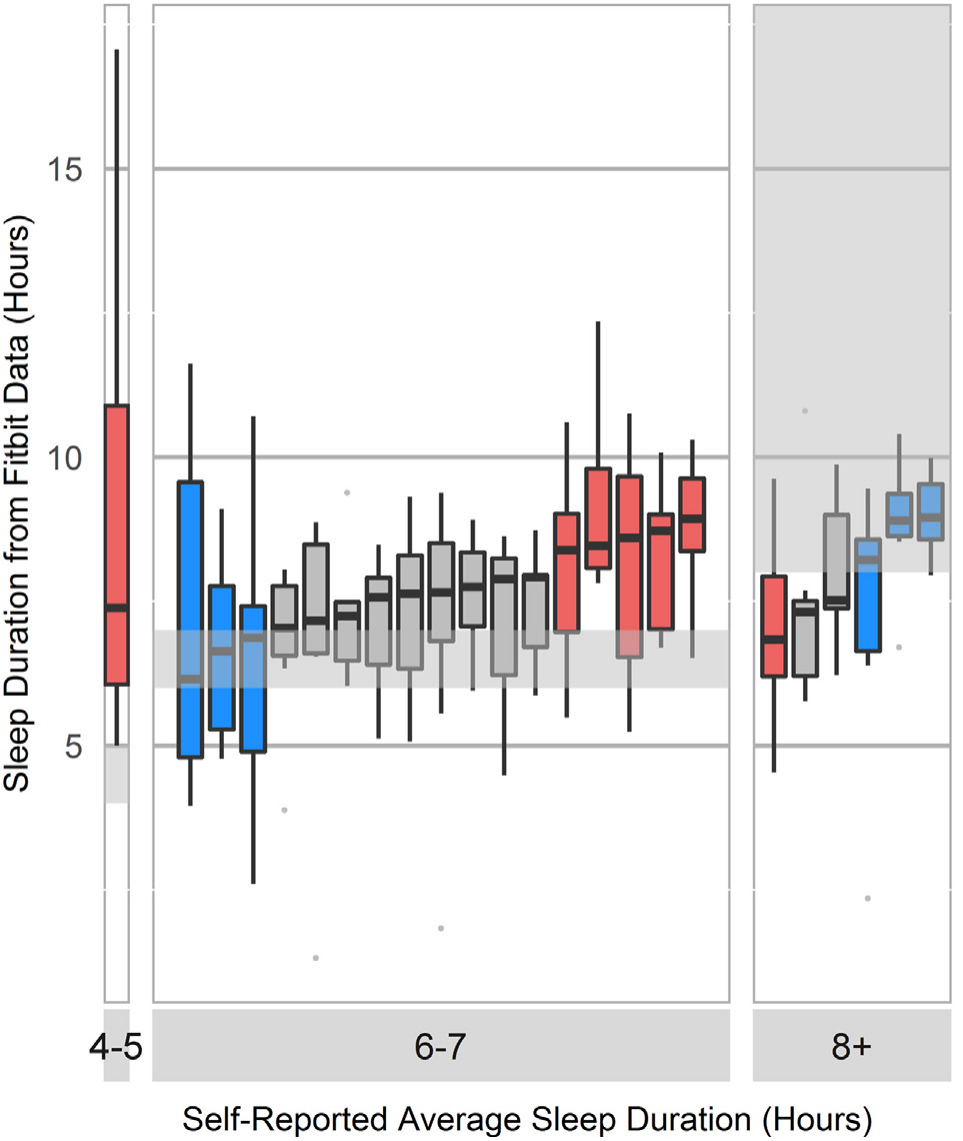
**Boxplot of self-reported sleep duration compared to Fitbit-reported sleep duration**. Survey response options included <2, 2–4, 4–5, 6–7, and 8+ h. Blue bars represent participants who selected the survey response that accurately described their average sleep duration, as verified by the wearable device. Red bars represent participants who selected an incorrect description of their average sleep duration on the survey, and gray bars represent users who selected one of two possible correct responses.

Participants systematically over- or underestimated their sleep habits (Figure 1). Since accurate sleep information is critical to modeling sleep-related circadian changes to mental acuity and performance, we implemented and trained model with objective sleep features gathered using the wearable device.

**Figure 2:**
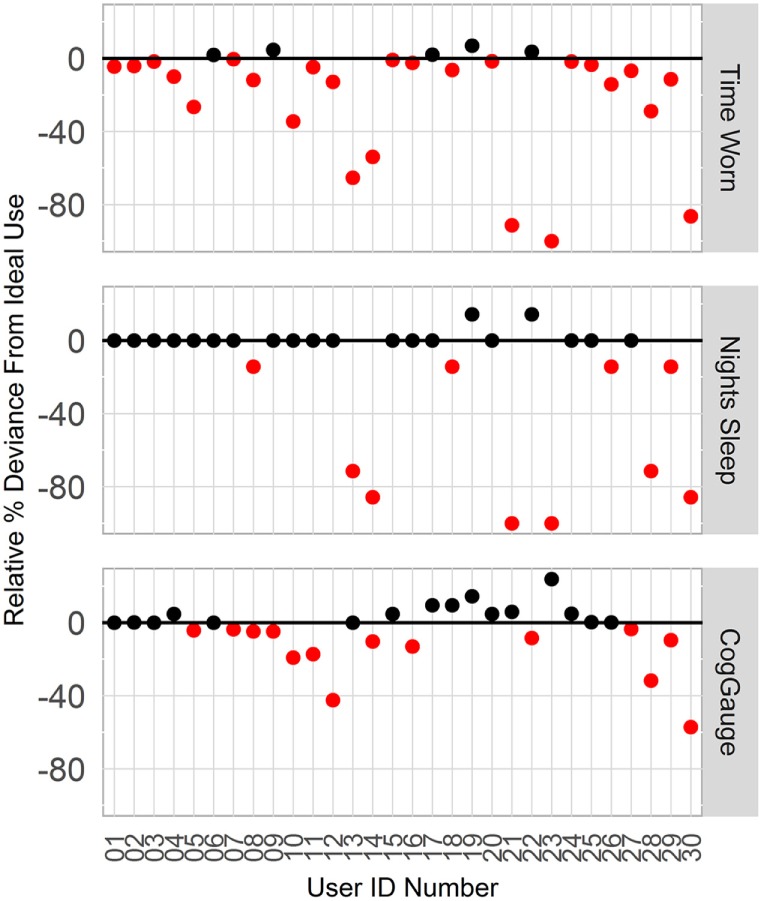
**Compliance with wearing the Fitbit device and responding to the CogGauge assessments among all 30 participants over the week long course of the experiment**. Six participants who did not wear the device, did not sync the device, or had a broken device were removed from model development.

Six participants who did not comply with instructions to wear the device and respond to the cognitive assessments, or who experience data loss were excluded from model development. Users A13 and A30 did not wear the device throughout the week; users A14, A21, and A28 experienced unexplained data loss; and user A23 had a broken device. User compliance is shown in Figure 2. Among all 30 participants, the percentage of time worn was 81.1 ± 30.0% of the ideal; night’s sleep was 87.8 ± 28.5% of ideal, and number of CogGauge sessions was 95.1 ± 16.2% of the ideal. Among the 24 participants used in model development, the percentage of time worn was 94.2 ± 9.49% of ideal; night’s sleep was 98.8 ± 7.19% of ideal, and number of CogGauge sessions was 96.8 ± 11.6% of the ideal. Although participants were allowed to play through the assessments at their convenience, dispersion of gameplay time was high, with only 74 of the 608 sessions recorded (12%) played within 3 h of the previous session. This afforded the model a snapshot of mental acuity throughout the day, allowing accurate training of the model.

**Figure 3:**
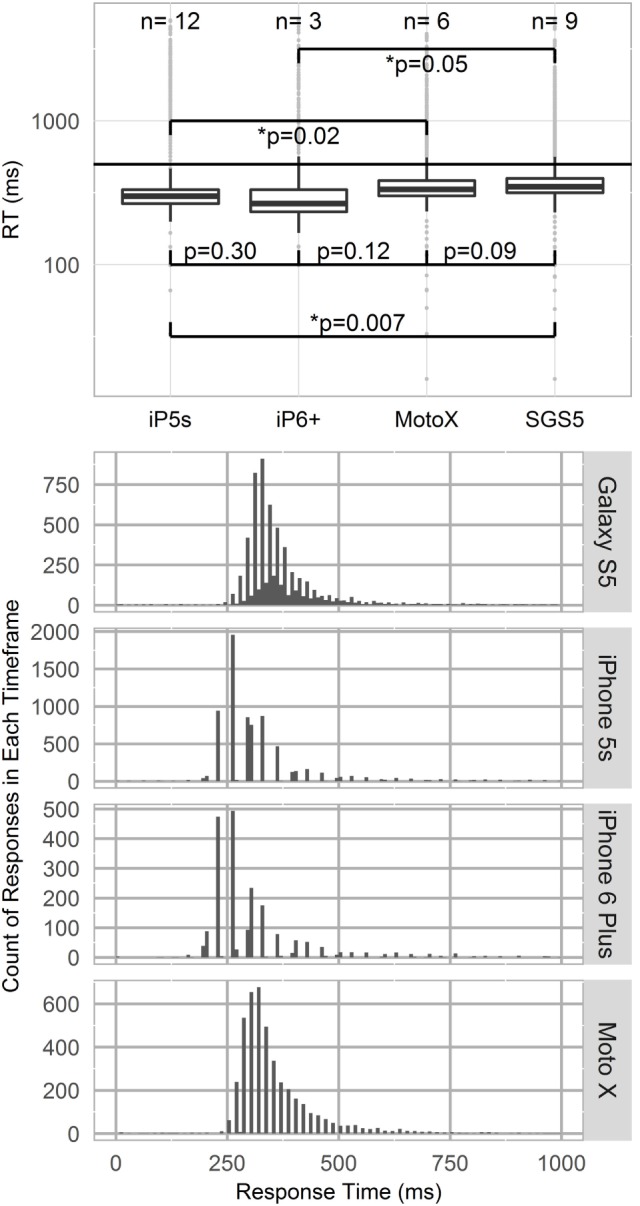
**Top panel—Logarithmic boxplot of psychomotor vigilance test (PVT) response times (RTs) by device type**. iPhone devices (iP5s, iP6+) recorded lower RTs than Samsung Galaxy S5 or MotoX devices. *p*-Values indicated between devices per Kruskal–Wallis testing. Standard PVT lapse threshold of 500 ms shown by horizontal line. Bottom panel—Phone RT discretization. Histograms of RT by phone model, with binwidths of 8.33 ms. All phone models show 60 Hz RT discretization.

Temporal effects were observed across devices (Figure 3). RT measures of central tendency and spread shifted across devices during the PVT. Kruskal–Wallis testing indicated that iPhone devices (iP5s, iP6+) recorded significantly lower RTs than SGS5 (SGS5; iP6+ *p* = 0.05; iP5s *p* = 0.007) or MotoX (iP5s *p* = 0.02). Additionally, phones were found to discretize their RTs. Figure 3 shows histograms of RT for each phone model with bin widths of 8.3 ms. Note that no space is plotted between bins. All phones show some responses discretized every 16.7 ms, which matches expected latency for a 60 Hz device.

**Figure 4:**
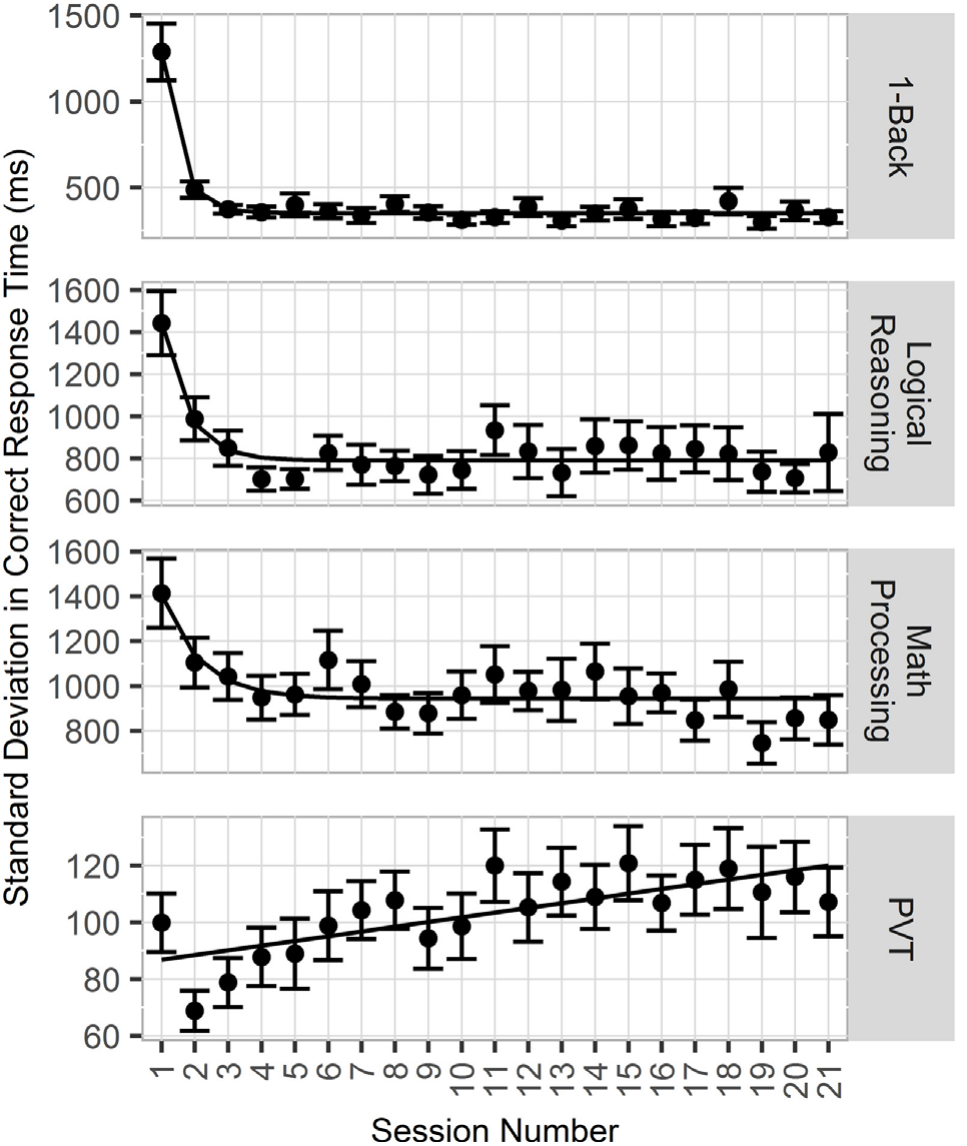
**Plot of effects of repeated exposure to CogGauge assessments on response time (RT)**. 1-Back, logical reasoning, and math processing RT followed an exponential decay relative to sessions played with half-lives of 0.67, 1.5, and 2.6 sessions, respectively.

For all CogGauge assessments except PVT, metrics such as RT and SD in CRT followed an exponential decay over time as participants were repeatedly exposed to the assessments. Figure 4 shows this effect. The half-lives for each assessment with a training effect were 0.67 CogGauge sessions for 1-Back, 1.5 sessions for logical reasoning, and 2.6 sessions for math processing. To eliminate these training effects from affecting the model, the first three CogGauge sessions, a value chosen to be greater than the largest half-life, were excluded from modeling.

The initial feature list was reduced with the goal of moderating the feature space to avoid overfitting, developing an uncorrelated feature space, and removing features whose value may vary with phone model or operating system. RT features and features with known correlations were removed, and remaining features were input into the exhaustive wrapper feature importance measurement. The final set of features selected for inclusion were chosen to have the lowest BIC while including metrics from multiple games and metrics from the PVT game to provide a broad definition of mental acuity and remain consistent with previous models. Table 4 shows the five feature combinations with the lowest BIC that matched the search criteria.

**Table 4:** **Top five combinations of input features with the lowest Bayes information criterion (BIC) values that both contain metrics from at least three games and at least one metric from psychomotor vigilance test (PVT)**.

Feature inputs	BIC
Logical reasoning CR% PVT SD in correct response time (CRT) 1-Back SD in CRT	−0.9

Logical reasoning CR% PVT SD in CRT Math processing timeout	0.2

Logical reasoning CR% PVT SD in CRT Math processing SD in CRT	0.7

Logical reasoning CR% PVT SD in CRT Math processing% correct	0.7

Logical reasoning CR% PVT SD in CRT 1-Back CR%	0.8

The final features selected for incorporation in the model were PVT SD in CRT, logical reasoning percent correct, and 1-Back SD in CRT. The mental acuity metric, an equally weighted linear combination of these metrics, was thus defined using Eq. 3:

(3) Mental acuity=13Logical Reasoning percent correct+(200*1003)(1PVT SD in CRT)+(600*1003)(11−Back SD inCRT)

**Figure 5:**
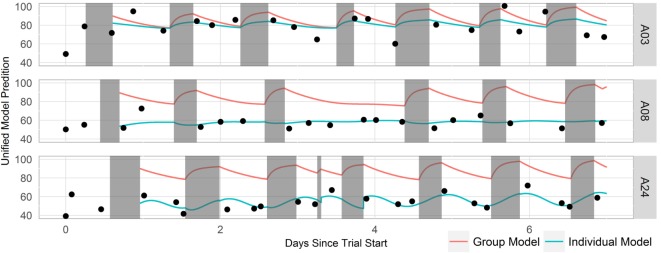
**Timeline of three representative participant’s mental acuity, sleep data, and model fit**. Mental acuity results are shown as black dots, sleep periods are shown in gray, the group model fit is shown as a red line, and the individual fits are shown as blue lines.

**Figure 6:**
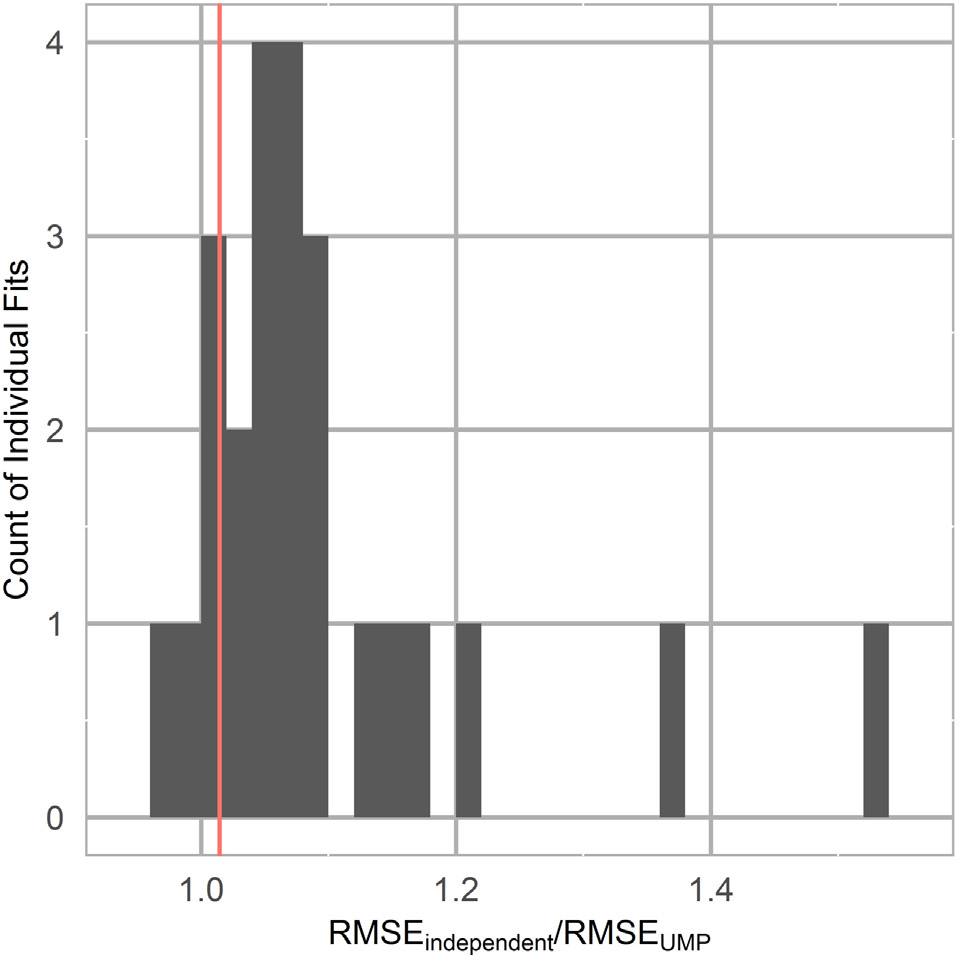
**Unified model of performance fit as measured by the ratio of the root mean squared error (RMSE) of the unified model fit to a horizontal line fit, representing mental acuity independent of sleep effects**. Group model is represented by a vertical line.

Group and individual least-squares fits to the unified model of performance using this mental acuity metric and cleaned Fitbit sleep data for three representative participants are shown in Figure 5. The mental acuity metric is associated with circadian fluctuation, with the highest mental acuity generally observed immediately following sleep and the lowest mental acuity observed before bed. The performance of the models, as measured by the fit quality, is shown in Figure 6. Individual models consistently performed better in modeling mental acuity results than the group model. Additionally, most models fit the mental acuity data better than a model independent of sleep effects and exceeded the fit quality of the unified model of performance for 19/24 participants.

## Discussion

The current study indicates the feasibility of creating an individualized, mobile assessment and prediction of mental acuity compatible with the majority of mobile operating systems. The custom mobile application, called SHARPR, joins a large number of other mobile applications focused on sleep (32) and cognitive training (33), but is unique in its ability to utilize sleep information to measure and predict temporal changes to cognitive abilities.

The current effort utilized the unified model of performance to model circadian changes to mental acuity due to time of day and sleep. The unified model of performance was developed to model changes to vigilance with high accuracy arising from total sleep loss or chronic sleep restriction (15) and has been utilized primarily in laboratory environments. In contrast, the current effort sought to utilize the unified model of performance to predict changes to mental acuity, including vigilance, working memory, and linguistic metrics for a more complete assessment of executive function. Participants were not asked to alter their sleep schedules, utilized a wearable device to monitor sleep, and responded to a mobile application to measure mental acuity.

Following recent military operations in the Middle East, a significant number of servicemen suffer negative health consequences as a result of military stressors, including changes to mental health (12). Sleep disturbance, including insomnia, sleep fragmentation, and nightmares has been shown to be a strong risk factor and predictor for mental health (34). Disruption of normal sleep routines affects an individual’s circadian rhythms, causes stress, cognitive decrements, and psychological effects. Sleep disturbances are common during military deployment, and the effect of such sleep disturbance on soldier mental acuity and subsequent changes to mental health should be taken into account prior to mission assignment.

An important finding of the current study relates to application development across mobile platforms, and that differences in hardware have the potential to significantly sway results dependent on temporal measures such as RT. Such alterations are expected to arise not only from differences in hardware and peripheral capabilities (35) but also background computing tasks (36) and even the method a user chooses to touch the screen (37). Due to such temporal differences observed across devices, feature selection for our mental acuity metric utilized features least likely to exhibit variability across devices. Additionally, PVT RT over 1 s were seen in this study. Unlike most previous work where subjects were monitored in a laboratory setting, in this study, participants were asked to respond to the cognitive assessments at their convenience. The longer RT is believed to be due to inattentive answers, when a participant is momentarily distracted from the assessment by an outside stimulus. Distracted answers were not included in the calculation of statistical values for PVT RT. Of note is the gradual increase in PVT RT observed over the course of the 1-week study. Such an observation has been described previously for the PVT, and may be due to continued exposure to a task considered boring or monotonous, resulting in a gradual decrease in mean RT reliability and increasing variability (38).

A number of previous efforts have focused on modeling cognition or performance based on self-reported sleep. Our data and others suggest that self-reported sleep is inaccurate (22). More objective options include polysomnography or actigraphy. There is a known bias toward overestimation of sleep duration inherent in actigraphy systems. A handful of previous studies compared Fitbit devices to other actigraphy sensors and polysomnography in general, and indicated that Fitbit sleep metrics are similar to other actigraphy devices, which, due to the prediction of sleep based on movement rather than gross neural activity, underperform compared to EEG (39–41). Similar reliability/validity data have been described for other Fitbit algorithms (42, 43).

Technical issues were encountered by a number of participants during the study, specifically with regard to the wearable device. We found that the Fitbit device began to overwrite stored data after approximately 5 days without syncing to the cloud, with sleep data overwritten before heart rate data. Thus the two users reported in this study with continuous heart rate information, indicative of wearing the device, with no sleep records was likely due to infrequent syncing of the device. Future software versions will include sync reminders and notifications of poor tracking to remove the need for manual sleep scoring. The users with intermittent heart rate data, but other data streams intact, are indicative of poor device placement, where accelerometer values are obtained but not heart rate *via* the included PPG sensor.

As most commercially available health-care apps remain untested (44, 45), we are currently evaluating the approach in the initial target population, active duty, and reserve US military personnel.

## Ethics Statement

This study was carried out in accordance with the recommendations of a series of Institutional Review Boards [Copernicus Group IRB, Durham, NC, USA; US Army Medical Research and Material Command Human Research Protection Office (HRPO), Fort Detrick, MD, USA] with written informed consent from all subjects. All subjects gave written informed consent in accordance with the Declaration of Helsinki. The protocol was approved by a series of Institutional Review Boards including Copernicus Group IRB and HRPO.

## Author Contributions

BW and NN designed the experiments. BW and KV analyzed the data. BW, NN, and KV wrote the manuscript.

## Disclaimer

The views, opinions and/or findings contained in this report are those of the authors and should not be construed as an official Department of the Army position, policy or decision.

## Conflict of Interest Statement

The authors declare that the research was conducted in the absence of any commercial or financial relationships that could be construed as a potential conflict of interest.
